# Exploring the Influence of Approximations for Simulating Valence Excited X-ray Spectra

**DOI:** 10.1021/acs.jpca.4c06150

**Published:** 2024-12-04

**Authors:** Thomas J. Penfold, Basile F. E. Curchod

**Affiliations:** † Chemistry - School of Natural and Environmental Sciences, Newcastle University, Newcastle upon-Tyne NE1 7RU, United Kingdom; ‡ Centre for Computational Chemistry, School of Chemistry, University of Bristol, Cantock’s Close, Bristol BS8 1TS, United Kingdom

## Abstract

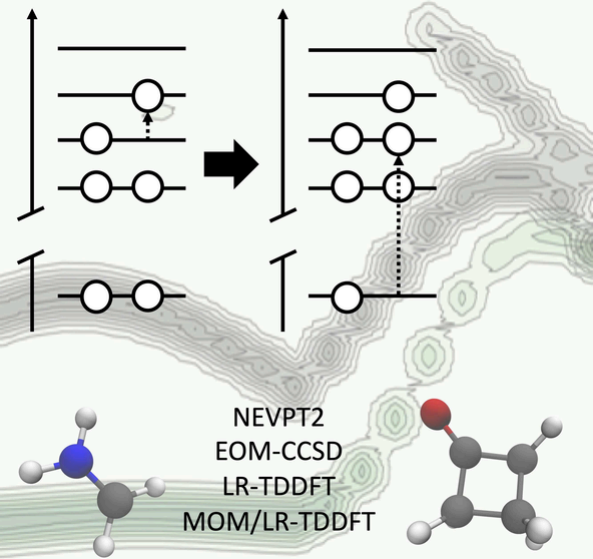

First-principles simulations of excited-state X-ray spectra are becoming increasingly important to interpret the wealth of electronic and geometric information contained within femtosecond X-ray absorption spectra recorded at X-ray Free Electron Lasers (X-FELs). However, because the transition dipole matrix elements must be calculated between two excited states (i.e., the valence excited state and the final core excited state arising from the initial valence excited state) of very different energies, this can be challenging and time-consuming to compute. Herein using two molecules, protonated formaldimine and cyclobutanone, we assess the ability of *n*-electron valence-state perturbation theory (NEVPT2), equation-of-motion coupled-cluster theory (EOM-CCSD), linear-response time-dependent density functional theory (LR-TDDFT) and the maximum overlap method (MOM) to describe excited state X-ray spectra. Our study focuses in particular on the behavior of these methods away from the Franck–Condon geometry and in the vicinity of important topological features of excited-state potential energy surfaces, namely, conical intersections. We demonstrate that the primary feature of excited-state X-ray spectra is associated with the core electron filling the hole created by the initial valence excitation, a process that all of the methods can capture. Higher energy states are generally weaker, but importantly much more sensitive to the nature of the reference electronic wave function. As molecular structures evolve away from the Franck–Condon geometry, changes in the spectral shape closely follow the underlying valence excitation, highlighting the importance of accurately describing the initial valence excitation to simulate the excited-state X-ray absorption spectra.

## Introduction

Time-resolved spectroscopy is a critical tool for understanding the ultrafast dynamics of matter.^1,2^ As the ability to perform increasingly complicated experiments is rapidly progressing, there is an ever greater need for theory and computation to disentangle the rich information content within the recorded spectra.^3−5^ The importance of computational spectroscopy is especially well showcased within X-ray spectroscopy,^6^ where the transformative effects of next-generation light sources are pushing the limits of the technique, facilitating new insights into the structure and dynamics of molecules and materials as well as opening up avenues across a wide range of research fields, including ultrafast time-resolved signals.^7−9^

Simulating excited-state absorption spectra requires the calculation of transition intensities between *excited* electronic states, often characterized by the corresponding oscillator strength,
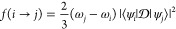
1with ψ_*i*_ and ψ_*j*_ representing the excited electronic wave functions for states *i* and *j*, ω_*j*_ – ω_*i*_ the energy gap between them and  is the dipole operator. In the optical regime, simulations must capture oscillator strengths between the valence excited electronic state of interest to the higher lying excited electronic state, which is usually between 2 and 4 eV. This range makes it straightforward to use wave function-based^10,11^ and density-based^12,13^ methods.

While conceptually similar, calculating intensities for excited-state absorption spectra into the X-ray regime poses the challenge of calculating the oscillator strength between core–hole and valence excited electronic states, which exist on vastly different energy scales and are separated by many intervening excited electronic states that are not involved in the transitions of interest.^6^ To address this issue, significant theoretical advances have been made in core–hole spectroscopy, often using the core–valence separation (CVS) approach.^14−16^ This technique assumes a block-diagonalization of the electronic Hamiltonian that decouples the valence and core excited subblocks, producing a cost-effective method that yields core excited states as the lowest energy solutions. While this strategy represents an efficient way of dealing with core excited states, it means that separate valence excited-state and core excited-state calculations are required and need to be subsequently coupled, as a single simulation within the core–valence separation approach will not typically include both the core excitations and the valence excitations that would be required.

An alternative approach is the multiconfigurational self-consistent field (MCSCF) family of methods, such as complete-active-space SCF (CASSCF)^17^ and restricted-active-space SCF (RASSCF),^18^ both of which can subsequently be improved via (second order) perturbation theory (PT2), arriving at the CASPT2,^19,20^ RASPT2^21^ and *n*-electron valence-state perturbation theory (NEVPT2)^22−24^ schemes. These methods, traditionally used for the ground electronic state or the valence excited electronic states, can be extended to the X-ray regime by including in their active space not only valence orbitals but also the core orbitals required to capture all of the important core and valence excitations.^25,26^ These methods have been used widely to simulate core–hole spectra of molecules in their ground^27−30^ and excited states.^31−36^ While they can be highly accurate, their computational expense increases substantially with the size of the active space due to the simultaneous inclusion of valence and core orbitals, meaning that an active space can become large even for comparably small molecules. In addition, for valence and core excitations obtained from separate calculations, one must couple nonorthogonal sets of optimized orbitals, necessitating a biorthogonalization procedure to arrive at convenient working equations for the transition densities.^26,37,38^ To address the computational expense, Neville et al.^41^ extended the DFT/MRCI approach^39,40^ to core excited states. This methodology retains a multireference character for the electronic wave function while preserving a scaling with system size similar to that of linear-response time-dependent density functional theory (LR-TDDFT),^42^ and was shown to be accurate for simulating the K-edge spectra for ground- and excited-state organic compounds.^41^

Alternatively, Coriani and co-workers^43−47^ have used coupled-cluster based approaches for simulating excited-state X-ray spectra, usually within the frozen core (fc) CVS-EOM-CCSD framework.^43^ Importantly and in line with all single-reference methods used to simulate excited-state X-ray spectra, this approach is based upon the assumption that the final core–valence excited states can be reached by core excitations from a ground-state reference electronic wave function. Consequently, the Hartree–Fock–Slater determinant, representing the ground state as the reference for the CCSD calculation, is used to separately calculate the initial valence excited and final core excited states using EOM-CCSD and fc-CVS-EOM-CCSD, respectively. The transition energies for core–valence excitations are subsequently computed as the energy differences between the final core states and the initial valence state. The oscillator strengths for the transitions between the two excited states are obtained from the transition moments between the EOM states according to the EOM-CC theory.^43,48,49^ Conceptually similar methods have also been performed using the algebraic diagrammatic construction (ADC)^50,51^ class of methods. The strategy underlying these approaches has the disadvantage that, since one of the valence electrons is already excited at the instant of X-ray absorption, many of such final excited states cannot be connected by one-electron excitation from the ground state, and core excitation from valence excited states can be dominated by configurations of double or higher excitation character relative to the ground-state reference. These configurations may not be fully described by using single-reference methods that include only single and double excitations. To somewhat overcome this, Tsuru et al.^52^ introduced the high-spin open-shell reference (HSOR) and low-spin open-shell reference (LSOR) CCSD approaches, inspired by the maximum overlap method (MOM),^53^ which previously combined with LR-TDDFT to calculate excited-state X-ray signals.^33,54^ Here, instead of using a ground-state reference wave function, the MOM method generates a reference that mimics the valence excited state of interest. However, the definition of this valence excited state is based upon a single excited determinant (e.g., HOMO → LUMO, with orbital relaxation) and consequently this approach is unsuitable for describing appropriately critical points on the excited-state potential energy surface with a multiconfigurational character.

An alternative computationally inexpensive approach of addressing the reference wave function and the final electronic states accessible has been introduced by Bannwarth et al.,^55^ the so-called hole–hole Tamm–Dancoff-approximated density functional theory (hh-TDA) method. This method starts from an artificial doubly negative (*N* + 2) electron reference state and constructs ground- and excited-state electronic wave functions by applying a pair of annihilation operators to this reference (recovering the neutral electronic states). The methodology was originally focused upon the efficient treatment of photochemistry of organic and biochemical systems that involve several low-lying excited states—particularly those with both low-lying ππ* and nπ*—but has been extended to treat ground and excited-state X-ray spectra.^56^ Through the construction of the (*N* + 2) electron reference wave function, the hh-TDA method is capable of describing excited electronic states, although only those involving transitions to the lowest unoccupied molecular orbital (LUMO). Mixed-reference spin-flip time-dependent density functional theory (MRSF-TDDFT) has also been used.^57^ In ref (58) the authors demonstrated that combining techniques such as MOM for building a relaxed core–hole particle reference and the same protocol with a double hole particle relaxation could produce accurate and computationally efficient K-edge spectra of core to valence hole excitation energies for ground and valence excited states. While MOM based approaches have been widely deployed for generating valence excited states or a core–hole particle relaxed reference wave function, we note that orbital-optimized DFT (OO-DFT) methods^59^ can also be used and have shown great promises in the treatment of core excited states.^60^

Given the aforementioned importance of simulating excited-state X-ray spectra and the recent progress in a variety of computational methods used to achieve it, in this work we use the photochemical pathways of protonated formaldimine^61,62^ and cyclobutanone^63,64^ to undertake a detailed study and assessment of the ability of *n*-electron valence-state perturbation theory (NEVPT2), equation-of-motion coupled-cluster theory (EOM-CCSD), linear-response TDDFT (LR-TDDFT) and the maximum overlap method (MOM) for simulating excited-state X-ray spectra. This test set permits a comparison between single-reference and multireference methods alongside simplifications for generating the initial valence excitation. We stress that there are two key limitations when considering the use of single-reference methods for the computation of X-ray absorption spectra of valence excited states, which will impact accuracy, namely, (i) the importance of doubly excited core excited final states and (ii) the potentially multireference nature of the initial valence excited state, which may become especially important away from the Franck–Condon region. The latter is much more understood considering the extensive literature on the accuracy of valence excited state, and consequently, this work places a larger focus upon the role of doubly excited core excited final states and the behavior and performance of these methods away from the Franck–Condon geometry, in the vicinity of important features on the excited-state potential energy surfaces, namely conical intersections.

## Methods and Computational Details

**Figure 1:**
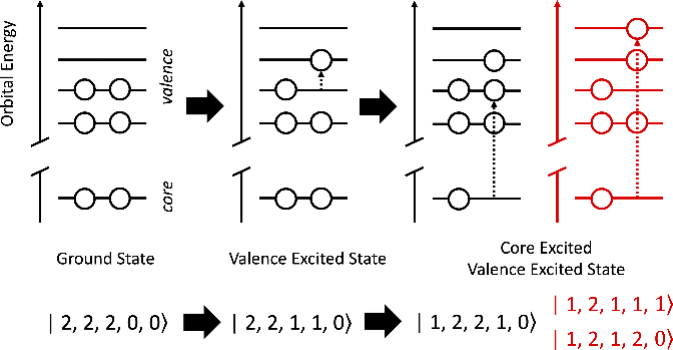
Scheme depicting a reference ground electronic state, a valence (e.g., HOMO → LUMO) excited electronic state, and a core excited valence excited electronic state. The configurations given under the scheme highlight the result of single excitation electron populations accessible at each step. Configurations in red highlight those not accessible within single-reference methods.

As discussed in the Introduction, single-reference methods (even if often more computationally efficient) are limited in simulating the final electronic states resulting from a core excitation from a valence excited state, as these final states are often dominated by configurations with a double or higher excitation character relative to the ground-state reference. To illustrate this and aid the discussion of the methods adopted in this work, Figure 1 shows a model system containing a 1s core orbital with two electrons and 4 valence orbitals, with the lower two orbitals containing two electrons: this starting initial state can be denoted |2, 2, 2, 0, 0⟩. Assuming a valence excited state of HOMO–LUMO character, this configuration becomes |2, 2, 1, 1, 0⟩. From this, a number of core excited states can be generated, including the three examples shown in Figure 1, namely, |1, 2, 2, 1, 0⟩, |1, 2, 1, 2, 0⟩ and |1, 2, 1, 1, 1⟩. While the first electronic configuration, |1, 2, 2, 1, 0⟩, could be easily described with a single-reference method, the other configurations highlight the complexity of the electronic states that can be generated in excited-state X-ray absorption spectroscopy.

NEVPT2 simulations^65,66^ were performed using ORCA 5^67,68^ and example input files are shown in the Supporting Information. Simulations commenced with a (state-averaged) SA-CASSCF simulation of the valence excited states. For protonated formaldimine, a (6,4) active space, including the two pairs of C–N σσ* and ππ* orbitals, was employed (see Figure S3). A cc-pVTZ basis set was used^69^ and simulations included all 40 valence excited states possible with this active space, calculated using state averaging. For cyclobutanone, a (6,5) active space, including the *n* orbital on the oxygen atom, the two ππ* and σσ* orbitals in the C–C_α_ bond (see Figure S4) was employed. A cc-pVDZ basis set was used^69^ and this simulation included all 50 valence excited states possible with this active space, calculated using state averaging. The SA approach performs a constrained minimization of a weighted sum over energies of multiple states, permitting a single set of optimized orbitals that are equally good for all electronic states considered. However, it has a couple of drawbacks, especially when large numbers of excited states are considered: (i) Excitation energies and oscillator strengths are dependent on the number of states and their corresponding weight for the averaging. (ii) It is not possible to describe electronic excitations out of the active model space.

Following the initial SA-CASSCF simulations, the active space was extended by 1 orbital (1s core) and 2 electrons and an additional 10 excited states (i.e., the core excited states) were included without any additional optimization of the orbitals. This new CASCI reference wave function was used as the starting reference for the NEVPT2 simulations, performed within the strongly contracted NEVPT2 (SC-NEVPT2) framework.^23,24,70^ As the CASCI approach incorporates all configurations within the active space chosen, all excited-state configurations are captured, provided the active space is judiciously chosen. Importantly, because CASCI do not reoptimize the orbitals in the calculation with the core excited states, these present calculations do not allow for orbital relaxation effects which can influence the accuracy of absolute excitation energies.

Excited-state X-ray spectra using LR-TDDFT were performed with ORCA 5,^67,68^ employing the PBE0 exchange and correlation functional^71,72^ and the same basis sets as the NEVPT2 simulations. Example input files are shown in the Supporting Information. An initial DFT calculation is performed, from which the core orbital of interest is rotated just below the valence orbitals of interest, and a LR-TDDFT calculation within the Tamm–Dancoff approximation^73^ is performed using a restricted excitation window incorporating the core and valence orbitals of interest. The transition dipole moment is calculated between all excited electronic states.^74^ We stress that this strategy remains within the linear-response regime throughout and therefore may differ from quadratic-response^75^ and second linear-response methods.^76^ The practical use of the adiabatic approximation also means that LR-TDDFT does not capture some of the final excited-state configurations shown in Figure 1 that differ by more than a single excitation from the reference ground state, e.g., |1, 2, 1, 2, 0⟩ and |1, 2, 1, 1, 1⟩.

The coupled-cluster calculations were performed using the Q-Chem quantum chemistry package.^77^ As shown by the example input file provided in the Supporting Information, these simulations use both an EOM-CCSD and an fc-CVS-EOM-CCSD calculation to determine the valence and core excited states from the ground-state Hartree–Fock reference. Once the manifold of these two sets of excited states has been calculated, the transition properties between them are obtained by using the approach described in ref (43). As both sets of states are calculated from the reference ground state, while the present method incorporates doubly excited determinants that have one excitation from a core orbital plus one excitation from a valence orbital, the coupling to the triple excitations would need to be included to accurately describe the doubly excited core excited states.

The MOM/LR-TDDFT hybrid approach^33,54^ (inputs shown in the Supporting Information) were performed with the Q-Chem quantum chemistry package^77^ using the PBE0 exchange and correlation functional.^71,72^ The MOM was used to generate the reference excited-state electronic wave function, using the dominant character at the Franck–Condon geometry for each molecule as a reference. For the first and second excited states of protonated formaldimine, this character corresponded to the HOMO–1 → LUMO and HOMO–2 → LUMO transitions, respectively. For the first excited state of cyclobutanone, this character corresponded to a HOMO–1 → LUMO transition. The MOM wave function was then used as the reference wave function for the subsequent LR-TDDFT within the Tamm-Dancoff approximation^73^ to produce the core excited states.

**Figure 2:**
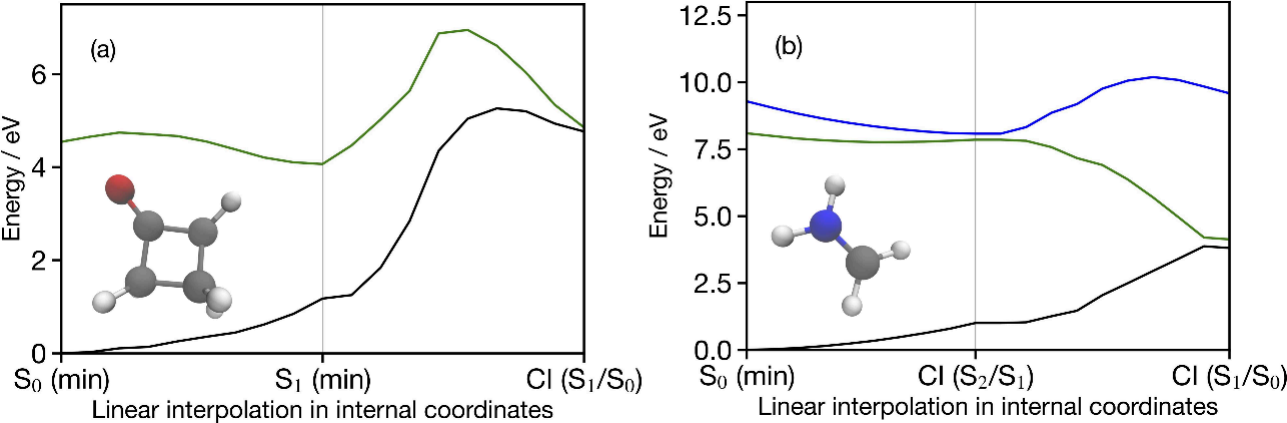
(a) Ground (black) and first (green) excited-state singlet potential energy curves obtained with NEVPT2 along a LIIC pathway for cyclobutanone from the optimized ground-state minimum to the S_1_ optimized minimum, and then to the S_1_/S_0_ conical intersection. (b) Ground (black) and first two excited-state (S_1_: green and S_2_: blue) singlet potential energy curves obtained with NEVPT2 along the LIIC pathway for protonated formaldimine from the optimized ground-state minimum to the S_2_/S_1_ conical intersection, and then to the S_1_/S_0_ conical intersection. The potential energy curves along these LIICs obtained with the other methods used in this work are shown in the Supporting Information.

Throughout this work, we used the optimized geometries for protonated formaldimine (S_0_ min, S_2_/S_1_ CI and S_1_/S_0_ CI) and cyclobutanone (S_0_ min, S_1_ min and S_1_/S_0_ CI) taken from refs (61) and (63), respectively. Potential energy curves are generated by using linear interpolations in internal coordinates (LIICs) between these critical geometries. Figure 2 shows the ground- and excited-state electronic energies calculated using NEVPT2 along these LIICs, while comparable potentials using the other methods are shown in the Supporting Information.

## Results

### Spectral Properties in the Franck–Condon Region

**Figure 3:**
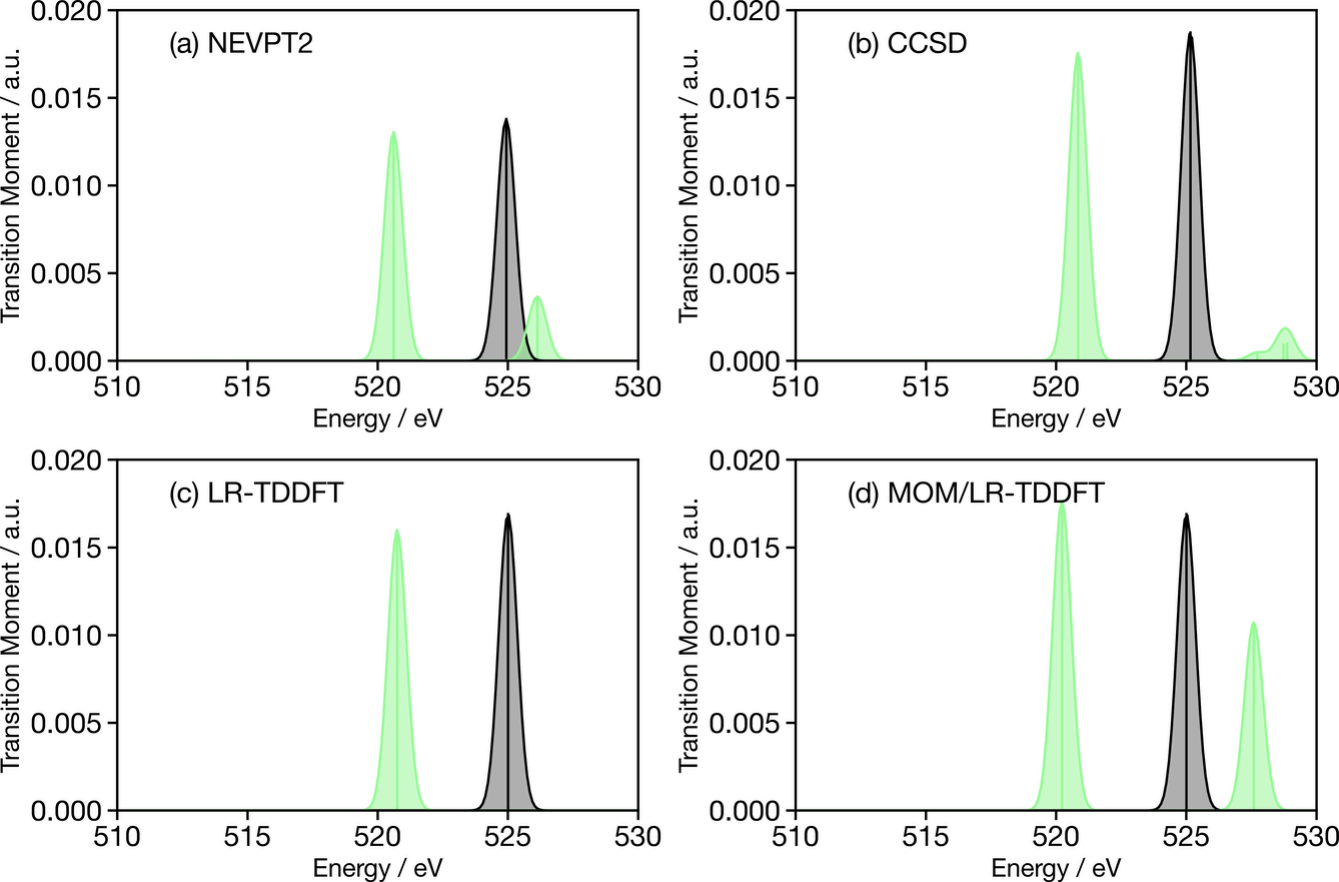
Oxygen K-edge X-ray spectra of cyclobutanone calculated for the ground-state optimized geometry with (a) NEVPT2, (b) CCSD, (c) LR-TDDFT and (d) MOM/LR-TDDFT. The black curve corresponds to the X-ray spectrum for cyclobutanone in its electronic ground state, while the green curve depicts the X-ray spectrum for the molecule in its S_1_ electronic state.

Figure 3a shows the ground-state (black) and valence excited S_1_ (green) oxygen K-edge X-ray spectra calculated at the ground-state optimized geometry of cyclobutanone using NEVPT2. In common with previous oxygen K-edge of ketones,^78^ a single transition corresponding to 1s → LUMO(C=O π*) is observed. When the molecule is in its excited S_1_ state HOMO *[n*(O)] → LUMO [C=O π*], this core–valence transition disappears due to the electronic character of the state and a new transition just above 520 eV is observed, corresponding to a transition from the 1s core to the hole in the HOMO [*n*(O)] generated by the excitation. The weak transition at 526 eV corresponds to a final state composed of double excitation involving a HOMO → LUMO and 1s → LUMO, which is significantly weaker than the 520 eV transition due to the dipole forbidden (nπ*) character of the HOMO → LUMO component.

For the EOM-CCSD calculations (Figure 3b), a similar trend emerges with a ground-state X-ray peak at 525 eV and an (S_1_) excited-state X-ray feature at 520 eV. However, the weak transition above 525 eV for the molecule in its S_1_ state cannot correspond to the same contributions as those observed in NEVPT2, as these multiple excitations mixing core–valence excitations to valence excitation cannot be captured using the EOM-CCSD formalism (see above). Instead, the characters of this transition are 1s → LUMO+2 and 1s → LUMO+4, both of which gain intensity from the fact that the valence excited state, although dominated by a HOMO → LUMO character, exhibit small contributions corresponding to a HOMO → LUMO+2 and HOMO → LUMO+4 character. These contributions are not visible in the NEVPT2 simulation, as these orbitals are not included within the active space.

Figure 3c shows the ground-state X-ray spectrum and the (S_1_) excited-state X-ray spectrum calculated by using LR-TDDFT(PBE0). The transitions 1s → LUMO(C=O π*, black) and 1s → HOMO (green) are in very good agreement with the NEVPT2 and coupled-cluster results. In contrast to the EOM-CCSD simulations, the higher energy weak transitions are not observed. This is because the S_1_ state in LR-TDDFT(PBE0) is of nearly pure HOMO → LUMO character and does not exhibit the other contributions giving rise to oscillator strength for these transitions in EOM-CCSD. In contrast, MOM/LR-TDDFT (Figure 3d) predicts a higher energy feature. In this case, the LR-TDDFT calculation is initiated from the excited MOM reference (with a HOMO → LUMO character) and is able to describe the final state observed in the NEVPT2 simulations exhibiting a combination of HOMO → LUMO and 1s → LUMO characters. However, this transition is much stronger in MOM/LR-TDDFT than in NEVPT2, because the valence excited state is a pure HOMO → LUMO (nπ*) transition, and therefore, the transition corresponding to the generation of the final state is 1s → LUMO. In the case of NEVPT2, there are weak contributions from other configurations which reduce the transition dipole moment.

In NEVPT2, this transition has dominant final character HOMO → LUMO and 1s → LUMO, with the former generated by the initial excitation. Consequently, in the one-electron picture, this is a 1s → LUMO transition from the valence excited S_1_ state. In NEVPT2, the transition dipole moment for the 1s → LUMO is slightly smaller than calculated using LR-TDDFT, as shown by comparing Figure 3a,c. However, this does not fully account for the difference. Indeed, in NEVPT2 the final state has both HOMO → LUMO and 1s → LUMO, with the former being very weak, as it is dipole forbidden. However, in MOM/LR-TDDFT, this transition is generated by the enforced occupancy, and therefore, its influence on the transition dipole moment is not taken into account. This has been expressed more clearly in the resubmitted manuscript.

**Figure 4:**
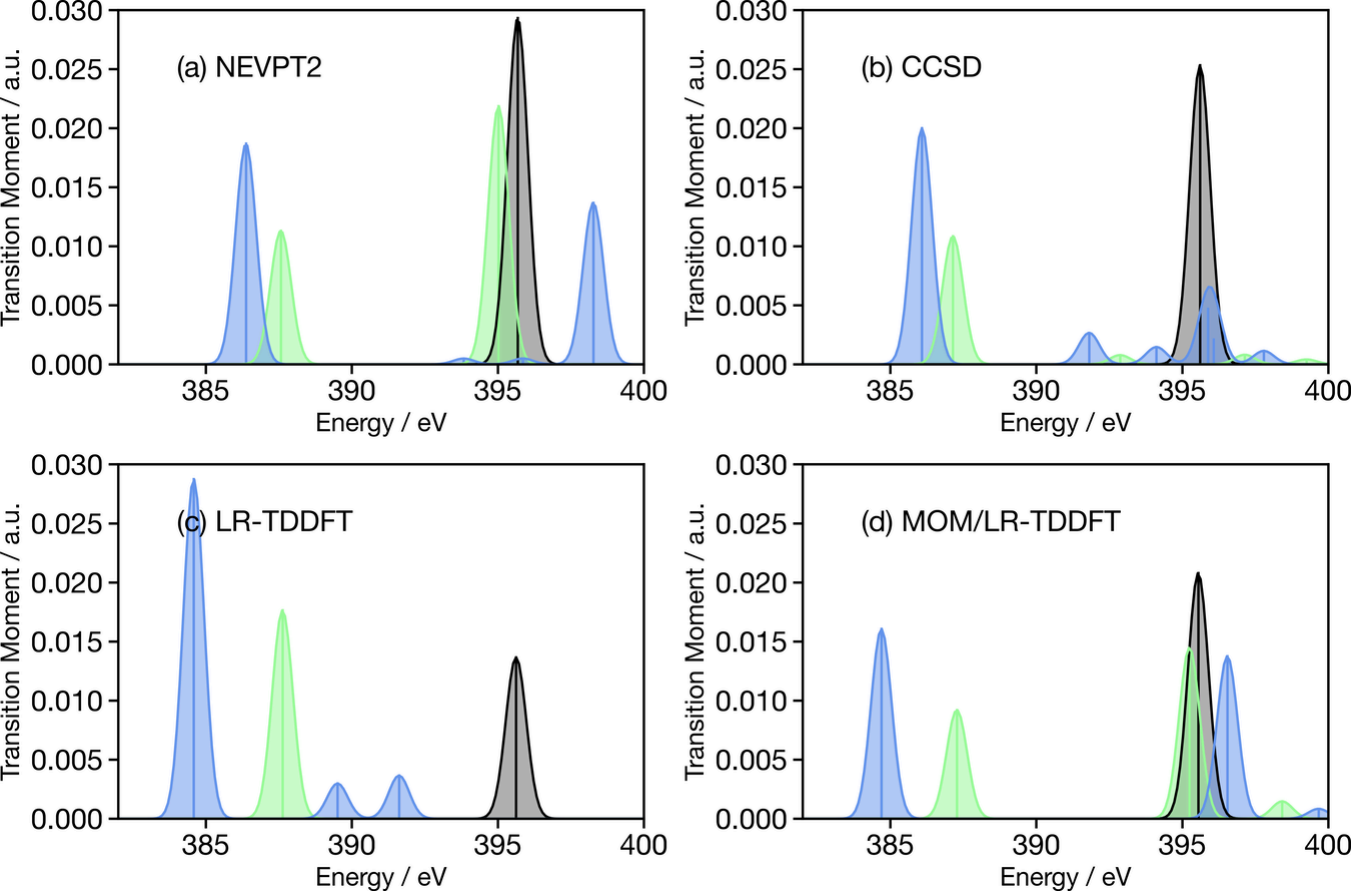
Nitrogen K-edge X-ray spectra of protonated formaldimine calculated for the ground-state optimized geometry with (a) NEVPT2, (b) CCSD, (c) LR-TDDFT and (d) MOM/LR-TDDFT. The black curve depicts the X-ray spectrum for protonated formaldimine in its electronic ground state, green curve the X-ray spectrum for the molecule in its S_1_ electronic state, and blue curve the X-ray spectrum for the molecule in its S_2_ excited electronic state.

Figure 4 shows the ground- and excited-state (both for S_1_ and S_2_) spectra at the nitrogen K-edge for protonated formaldimine at its optimized ground-state geometry. The ground-state X-ray spectrum shows a strong single peak (within the energy range of interest) at 395 eV corresponding to a 1s → LUMO (π*) transition. Similar to the observations made for cyclobutanone, each of the excited-state X-ray spectra shows transitions into the hole (HOMO–1 or HOMO–2 for the molecule in its S_1_ or S_2_ state, respectively) created by the valence excitation. The energy separation between these peaks is larger for LR-TDDFT and MOM/TDDFT, which is due to an overestimation of the energy gap between these valence excitations (as shown by the potential energy curves in Figure S2), reflected in the core excitations. Transitions at energies ≥392 eV correspond to higher order excitations. For these transitions, there is good correspondence between the results of NEVPT2 and MOM/LR-TDDFT, as observed for cyclobutanone, while the coupled-cluster simulations and LR-TDDFT are in between agreement.

Overall, this section demonstrates that, in the Franck–Condon region, the most significant excited-state X-ray spectral feature corresponds to the transition into the empty hole generated by valence excitation. As the final state can be represented as a single excitation from a ground-state reference, the corresponding transition can be accurately obtained with all of the methods tested herein. At higher transition energies, differences begin to appear due to either a transition missing as a result of a limitation in the orbitals present in the active space (NEVPT2) or the choice of the reference state (EOM-CCSD and LR-TDDFT). We will now move beyond the Franck–Condon region to investigate the performance of these methods in more detail, following the X-ray spectral features along LIICs connecting critical (valence) excited-state geometries and focusing especially on the low-energy features.

### Spectral Properties beyond the Franck–Condon Region

**Figure 5:**
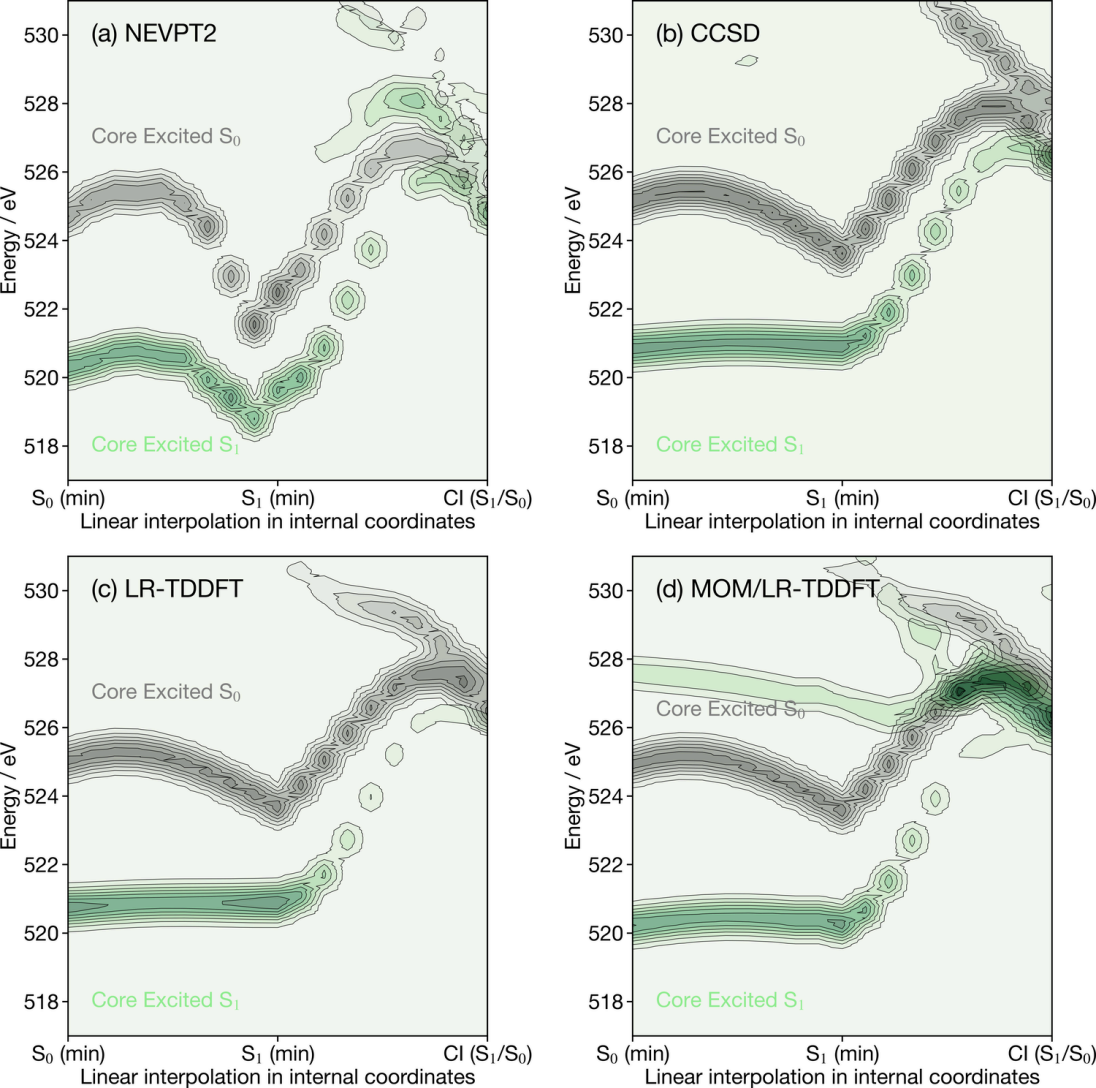
Oxygen K-edge X-ray spectra of cyclobutanone calculated with (a) NEVPT2, (b) CCSD, (c) LR-TDDFT and (d) MOM/LR-TDDFT along the LIIC pathway connecting the geometries representative of the ground-state (S_0_) minimum, the S_1_ minimum and the S_1_/S_0_ conical intersection. Black corresponds to the X-ray spectrum for the molecule in its electronic ground state, while green corresponds to the X-ray spectrum for the molecule in its S_1_ state.

Figure 5 shows the oxygen K-edge X-ray spectra for the ground state (black) and valence excited state S_1_ (green) of cyclobutanone, calculated along the LIIC pathway bridging the optimized ground-state minimum, the S_1_ minimum and the S_1_/S_0_ conical intersection. For each geometry, the X-ray signal obtained for each individual geometry (electronic energies and corresponding oscillator strengths) was broadened by using a Gaussian function (with a width of 1.0 eV) centered at each electronic energy and with a magnitude proportional to the oscillator strength; the final plot is obtained by combining the broadened X-ray signal for each geometry and (final) excited state considered. The main features of the X-ray signal are comparable between all four methods, with EOM-CCSD, LR-TDDFT and MOM/LR-TDDFT exhibiting trends in especially good agreement. In all four cases, the X-ray signals for the molecule in its ground electronic state and in its S_1_ state meet at the conical intersection geometry, and each spectral evolution closely follows the shape of the ground and S_1_ excited-state potential energy curves shown in Figure S1. Despite this general agreement among the methods, there are two key differences worth noting. First, the S_1_ excited-state X-ray signals predicted by CCSD, LR-TDDFT and MOM/LR-TDDFT appear to be largely independent of the underlying molecular geometry along the part of the LIIC connecting the S_0_ minimum to the S_1_ minimum, in stark contrast with the result of NEVPT2. Second, the spectral changes obtained with NEVPT2 appear to be much larger than the shifts in electronic energy observed in Figure S1.

**Figure 6:**
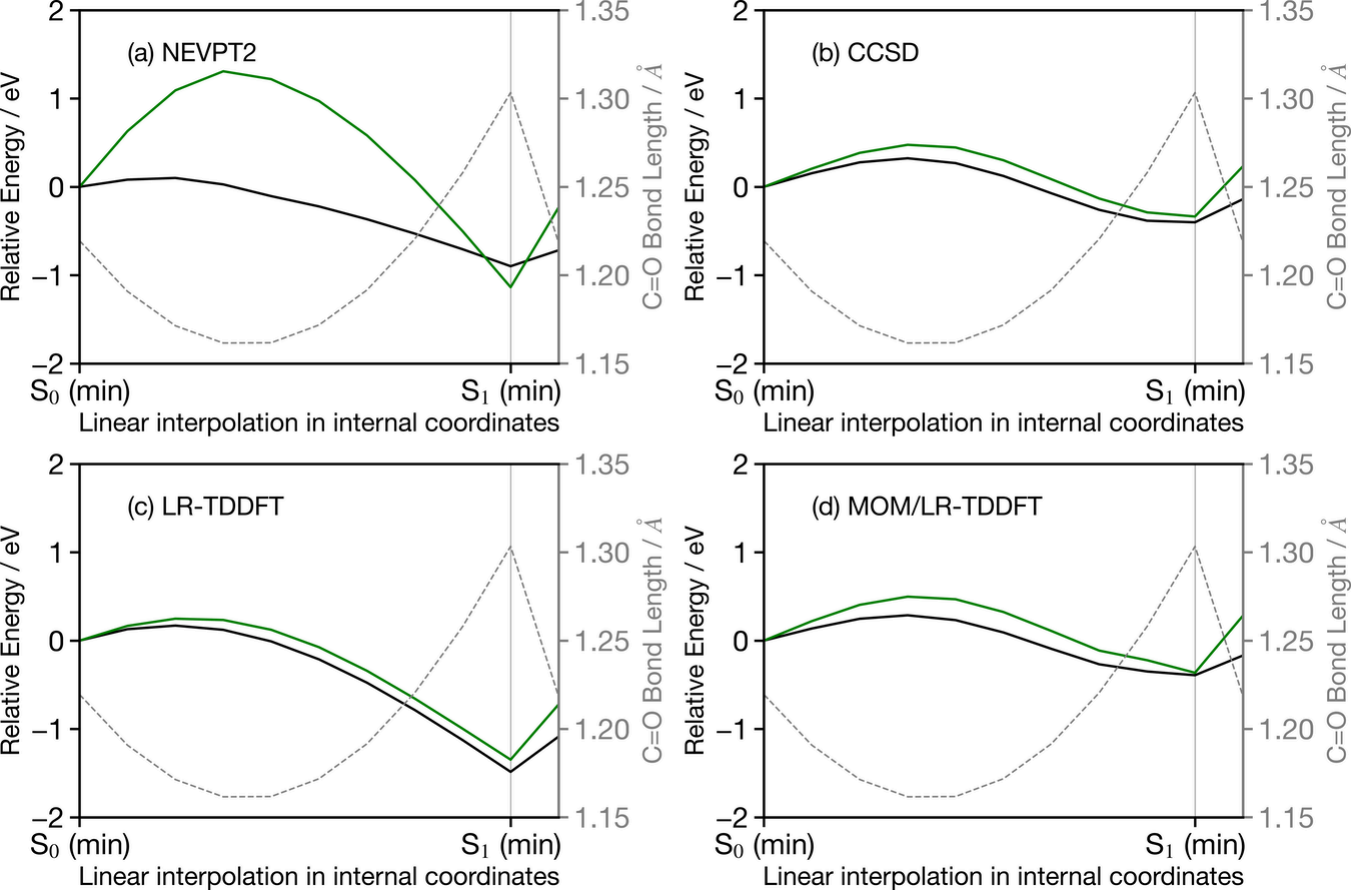
Relative electronic energies of the valence S_1_ (HOMO → LUMO, black) and lowest core (1s → LUMO, green) excited state along the LIIC pathway connecting the geometries representative of the ground-state (S_0_) minimum and the S_1_ minimum. All energies are relative to the S_1_ electronic energy in the Franck–Condon geometry. The gray dashed line shows the C=O bond length.

The first observation can be explained by the nature of the excitations involved (Figure 6). The lowest band, i.e., core excited S_1_ state (Figure 5), couples the S_1_ (HOMO → LUMO) valence excited state to the core excited state exhibiting a 1s → LUMO character (with respect to ground-state reference); as stated above, the core excitation fills the hole in the HOMO created by the valence excitation. The primary geometrical change along the LIIC pathway connecting the S_0_ (min) to the S_1_ (min) is an elongation of the C=O bond. This geometrical distortion does not change the energy of the HOMO (lone pair on the oxygen) significantly, and therefore, the shape of the S_1_ electronic energy along the LIIC is mostly driven by changes in the energy of the LUMO. In a single-particle picture, the core transition from this S_1_ excited state is of the type |2, 2, 1, 1, 0⟩ → |1, 2, 2, 1, 0⟩ (as per the notation used in Figure 1). Consequently, the transition energy in this single-particle energy should be sensitive to the energy of the 1s(O) or the oxygen lone pair (HOMO), both of which do not change their energy substantially along this portion of the LIIC pathway. As a result, the single-particle picture predicts that the energy of the core excited-state transition should not vary along the LIIC pathway, as observed for EOM-CCSD, LR-TDDFT and MOM/LR-TDDFT. This behavior is further detailed in Figure 6, which shows that the S_1_ valence (HOMO → LUMO) excitation and the core excitation (1s → LUMO) exhibit the same energy dependence with the molecular geometry (due to the sensitivity of the LUMO orbital to the C=O bond length), meaning that the change of the core excited S_1_ band (core excitation - valence excitation) does not change with geometry.

While the S_1_ excited energies (black curve in Figure 6) obtained with NEVPT2 exhibit a similar behavior along the LIIC pathway as the other methods, the core excitation shifts by ∼2 eV along the pathway, a value greater than the change in the S_1_ valence electronic energy observed in Figure S1 and responsible for the shape observed in Figure 5a. This strong variation of core excitation energy as a function of the nuclear geometry is consistent with previous work by Lundberg and co-workers^28,79^ for X-ray emission spectroscopy. Furthermore, the authors of this work also demonstrated that this sensitivity was significantly reduced in the case of single-reference methods, consistent with our observations for EOM-CCSD, LR-TDDFT and MOM/LR-TDDFT (shown in Figure 6b–d, respectively). Consequently, the behavior presented here for the core excited transition, which reflects Natoli’s rule^80^ (i.e., *Er*^2^ = constant, where *r* is the bond length and *E* is the absorption energy) appears to require a mixing of configuration only adequately described in a multireference method.

**Figure 7:**
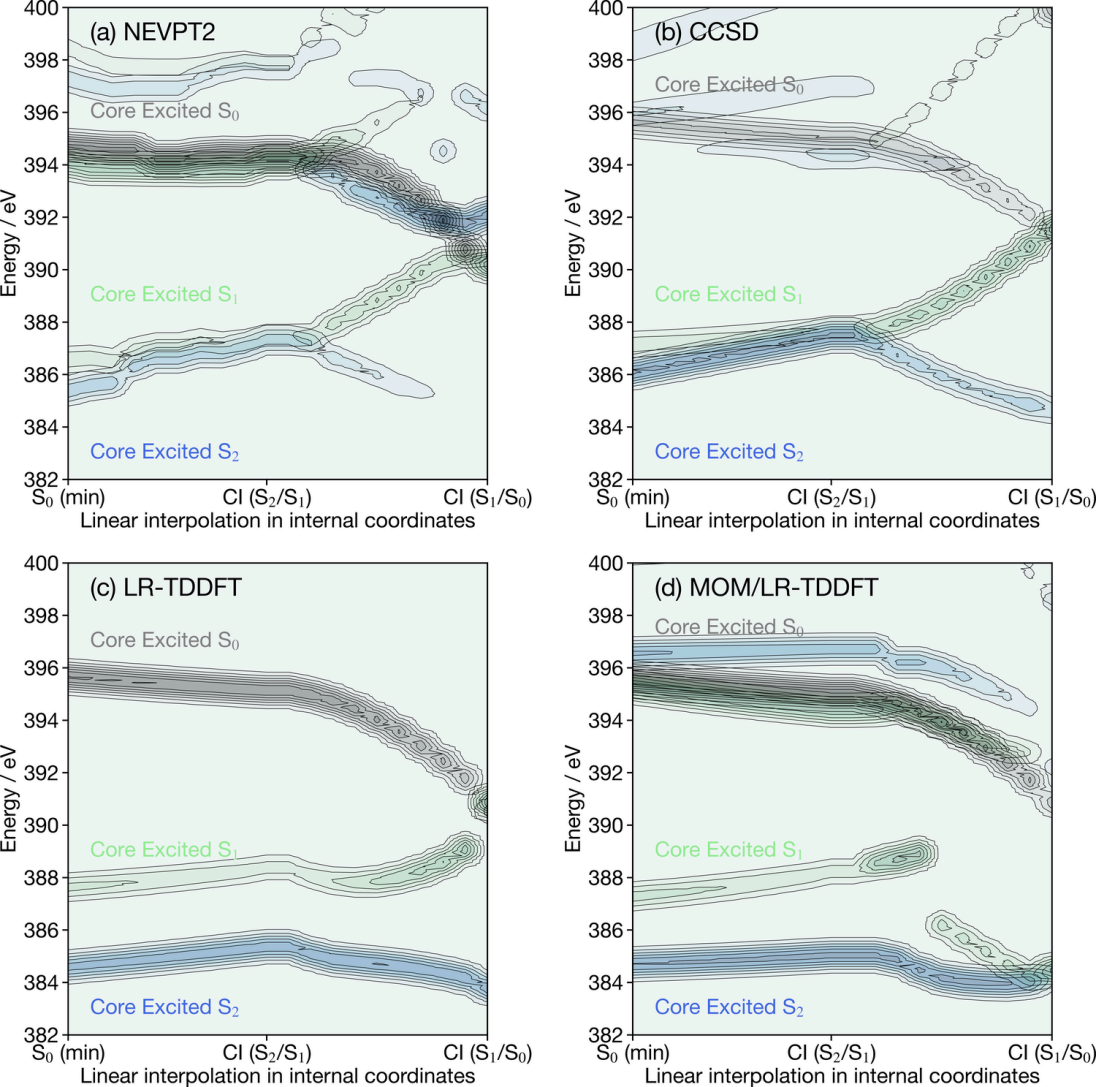
Nitrogen K-edge X-ray spectra of protonated formaldimine calculated using (a) NEVPT2, (b) CCSD, (c) LR-TDDFT and (d) MOM/LR-TDDFT along the LIIC pathway connecting the geometries representative of the ground-state (S_0_) minimum, the S_2_/S_1_ conical intersection and the S_1_/S_0_ conical intersection. Black corresponds to the X-ray signal for the molecule in its electronic ground state, green is the X-ray signal for the molecule in its S_1_ state, and blue is the X-ray signal for the molecule in its S_2_ state.

Figure 7 shows the ground-state (black) and valence S_1_ (green) and S_2_ (blue) excited nitrogen K-edge X-ray spectra of protonated formaldimine calculated along the LIIC pathway connecting the optimized ground-state geometry to the S_2_/S_1_ conical intersection and then the S_1_/S_0_ conical intersection. In contrast to that of cyclobutanone, significant differences are observed. For NEVPT2 and CCSD, the low-energy excited-state features are in very good agreement, and the most significant differences are the additional intense excited-state transitions which overlap with the ground-state transition and correspond to multiple electron transitions, as highlighted in the previous section. These transitions are stronger than the ones observed in the case of cyclobutanone, because the S_2_ (HOMO–2 → LUMO) transition is dipole allowed in contrast to the S_1_ (HOMO → LUMO) transition in cyclobutanone. The excited-state X-ray features along the LIIC pathway reflect the behavior of the excited-state electronic energies (shown in Figure S2). In contrast, significant differences are observed for the X-ray features calculated along the LIIC pathway with LR-TDDFT (Figure 7c) and MOM/LR-TDDFT (Figure 7d). As shown in Figure S2, these differences arise primarily from the different behavior of the LR-TDDFT-based electronic energies along the LIIC in the vicinity of the S_2_/S_1_ conical intersection (the geometry of the S_2_/S_1_ conical intersection is significantly different from that of a multireference method, as highlighted in ref (61).). This observation highlights the fact that the accuracy of electronic structure methods in describing adequately the valence excitations is likely to be a dominant component when calculating excited-state X-ray spectra.

## Discussion and Conclusions

The proliferation of high-brilliance short-pulse light sources such as X-FELs means that it is increasingly possible for X-ray spectroscopy to deliver highly detailed information about the local geometric and electronic structure of matter on the atomic scale of time, i.e., femtosecond. As a result, there is a clear need for computational strategies to simulate excited-state X-ray spectra to facilitate a detailed interpretation of the experimental data into the structure and dynamics of molecules and materials. To this end, this work has used protonated formaldimine and cyclobutanone as model molecular systems to undertake a detailed study and assessment of the ability of NEVPT2, EOM-CCSD, LR-TDDFT and MOM/LR-TDDFT for simulating excited-state X-ray spectra in the Franck–Condon region and beyond.

These simulations confirm that the primary feature appearing in the excited X-ray spectra is the transition from a core orbital to the hole generated by the initial photoexcitation, e.g., |2, 2, 2, 0, 0⟩ → |2, 2, 1, 1, 0⟩ → |1, 2, 2, 1, 0⟩, using the simple model discussed earlier. As this final excited state differs from the initial ground-state reference by only a one-electron (core to valence) transition, it is accurately captured by all methods listed above. All X-ray spectra also exhibit higher energy features whose intensity depends on the precise nature of the electron configurations involved. These features all exhibit contributions from higher order excitations, i.e., those which differ by more than one electron transition from the reference ground state. Consequently, they will only be captured by active-space methods (given that the adequate orbitals are in the active space) or those, such as MOM, which do not use a ground-state wave function as a reference. We do observe other transitions within the energy range considered with methods based on a ground-state reference (i.e., LR-TDDFT and EOM-CCSD), but these transitions gain intensity from configuration mixing with the one-electron transitions and remain therefore weak. Other transitions associated with promoting the 1s electron to other, higher virtual orbitals will become important but are not captured by single-reference methods, as they differ by more than one-electron excitation from the ground-state reference. This observation means that great care has been taken in interpreting excited-state features at energies close to and higher than the original lowest ground-state feature when using methods based on a ground-state reference.

Away from the Franck–Condon region, our calculations show that the shape of the excited X-ray spectral feature associated with filling the hole generated by the initial photoexcitation primarily reflects the shape of the underlying potential energy surface. High-energy features, beyond the scope of the present work may, due to selection rules, reflect different dynamics.^81,82^ Consequently, the evolution of the X-ray signal with the nuclear geometry will only be accurate if the underlying electronic-structure method is able to properly capture the valence excited electronic state. This issue is highlighted in the present work with the case of LR-TDDFT and MOM/LR-TDDFT for protonated formaldimine. For LR-TDDFT, the issue arises due to a difference in the shape of the underlying potential energy curve and, as highlighted in Figure S3, good agreement is achieved when using DFT/LR-TDDFT optimized geometries, consistent with the conclusions of ref (61). However, as the XMS-CASPT2 and LR-TDDFT/PBE0 geometries^61^ differ by ∼0.1 Å at the critical S_2_/S_1_ conical intersection geometry, an interpretation of an X-ray signal using different electronic-structure methods could lead to diverging conclusions about the structural dynamics. For MOM/LR-TDDFT, the issue arises from the inability of the method to accurately describe the valence excited states by using a single configuration/transition. The mixing of configurations becomes increasingly important away from the Franck–Condon region, especially in regions of the nuclear configuration space where topological features such as conical intersections appear; caution has to be exercised in applying these approaches in such regions.

Overall, this work highlights the advantages and limitations of the most commonly used strategies to simulate excited-state X-ray spectra. Future work in this area will focus on extending these studies to more complex systems, such as transition metal complexes^31,32,83,84^ or organic semiconductors^85,86^ which have been studied at X-FELs or laboratory sources based upon High Harmonic generation.

## Supporting Information Available

Ground and excited state potential energy surfaces for NEVPT2, EOM-CCSD, LR-TDDFT and MOM-LR-TDDFT, active space orbitals used in the NEVPT2 simulations, and example input files for all simulations (PDF)
