# Supplementary material for: Exploring the Influence of Approximations for Simulating Valence Excited X-ray Spectra
Source: J Phys Chem A. 2024 Dec 4;128(50):10826–36. doi: 10.1021/acs.jpca.4c06150 (PMC11664592; doi:10.1021/acs.jpca.4c06150)
Supplement: Supplementary file 1 — jp4c06150_si_001.pdf [file jp4c06150_si_001.pdf]

Supporting Information: Exploring the Influence of Approximations for Simulating Valence Excited X-ray Spectra

Thomas J. Penfold[*,†] and Basile F.E. Curchod[‡]

†*Chemistry - School of Natural and Environmental Sciences, Newcastle University, Newcastle upon-Tyne, NE1 7RU, United Kingdom*

‡*Centre for Computational Chemistry, School of Chemistry, University of Bristol, Cantock's Close, Bristol BS8 1TS, United Kingdom*

E-mail: tom.penfold@ncl.ac.uk

List of Figures

S1 Ground and first excited state singlet potential energy curves for NEVPT2 (a), EOM-CCSD (b), LR-TDDFT(PBE0) (c) and MOM/LR-TDDFT(PBE0) (d) along the linear interpolation in internal coordinates for cyclobutanone from the optimised ground state to the S_1 minimum and then S_1/S_0 conical intersection. S3

S2 Ground and first two excited state singlet potential energy curves for NEVPT2 (a), EOM-CCSD (b), LR-TDDFT(PBE0) (c) and MOM/LR-TDDFT(PBE0) (d) along the linear interpolation in internal coordinates for protonated formaldimine from the optimised ground state to the S_2/S_1 conical intersection and then S_1/S_0 conical intersection. S4

S3 Orbitals employed in the active space of protonated formaldimine S5
S4 Orbitals employed in the active space of cyclobutanone S5

Ground and Excited State Potentials

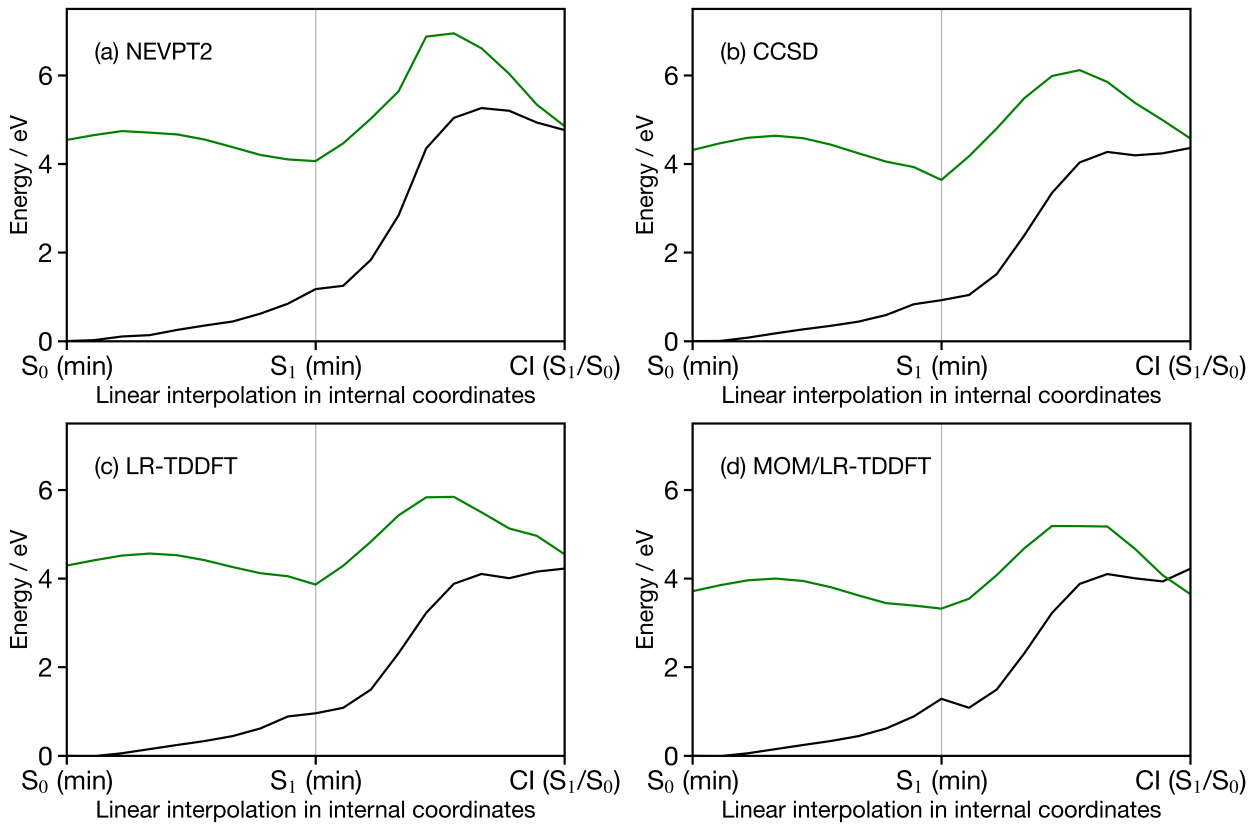

Figure S1: Ground and first excited state singlet potential energy curves for NEVPT2 (a), EOM-CCSD (b), LR-TDDFT(PBE0) (c) and MOM/LR-TDDFT(PBE0) (d) along the linear interpolation in internal coordinates for cyclobutanone from the optimised ground state to the S_1 minimum and then S_1/S_0 conical intersection.

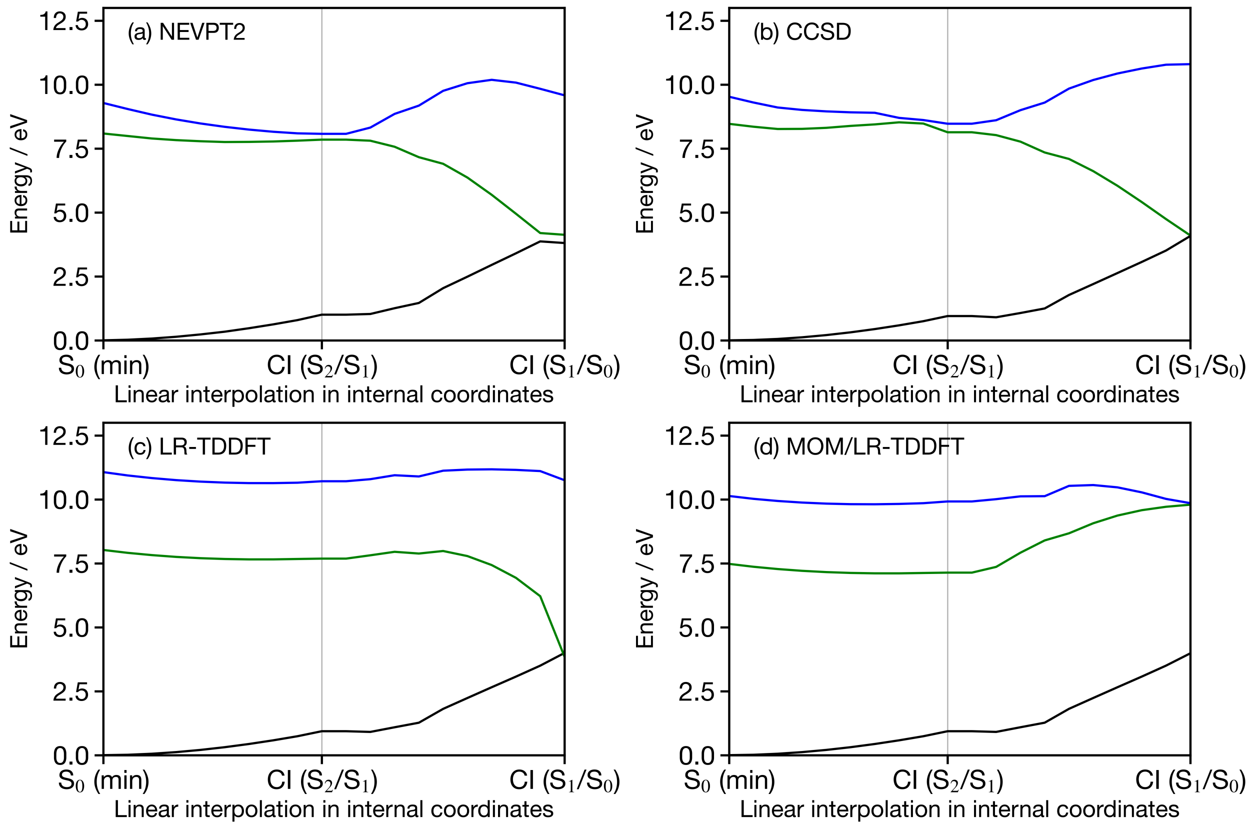

Figure S2: Ground and first two excited state singlet potential energy curves for NEVPT2 (a), EOM-CCSD (b), LR-TDDFT(PBE0) (c) and MOM/LR-TDDFT(PBE0) (d) along the linear interpolation in internal coordinates for protonated formaldimine from the optimised ground state to the S_2/S_1 conical intersection and then S_1/S_0 conical intersection.

Active Space Orbitals

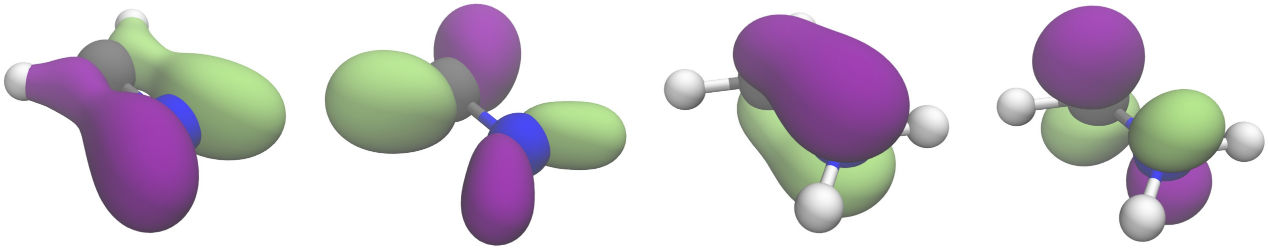

Figure S3: Orbitals employed in the active space of protonated formaldimine

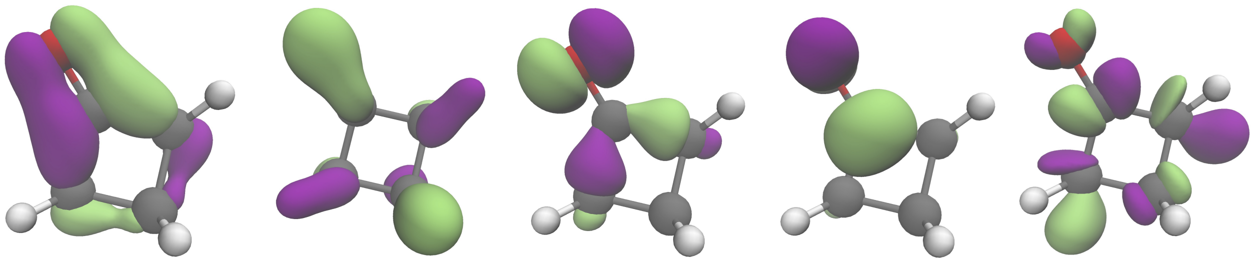

Figure S4: Orbitals employed in the active space of cyclobutanone

Example Input Files

NEVPT2 (ORCA)

```
!cc-pVTZ TightSCF nofrozencore

%casscf
 nel            6
 norb           4
 mult           1
 nroots         50
 MaxIter        1500
end

*xyzfile 1 1 input.xyz

$new_job

!cc-pVTZ TightSCF
!MORead NoIter nofrozencore

%scf
rotate {0,4,90} end end

%casscf
nel 8
norb 5
mult 1
nroots 14
maxiter 1
rel
 DoSOC true
 XESSOC true
 XASMOs 4
end
PTMethod SC_NEVPT2
end

*xyzfile 1 1 input.xyz
```

LR-TDDFT (ORCA)

```
!PBE0 cc-pVTZ TightSCF

*xyzfile 1 1 input.xyz

$new_job

!PBE0 cc-pVTZ TightSCF
!MOREAD NoIter

%scf
rotate {0,4,90} end end

%tddft OrbWin[0] = 4,7,8,12
       NRoots 90
       IRoot 1
       DOTRANS TRUE
end

*xyzfile 1 1 input.xyz
```

CCSD (Qchem)

```
$molecule
   1 1
{elements, x, y, z}
$end

$rem
   SYMMETRY = false
   SYM_IGNORE = true
   METHOD =  eom-ccsd
   BASIS =  cc-pVTZ
   SCF_CONVERGENCE =  8
   EE_SINGLETS =  5
   N_FROZEN_CORE =  1
   CVS_EE_STATES =  10
   CC_TRANS_PROP =  2
$end
```

MOM/LR-TDDFT (Qchem)

```
$molecule
1 1
{elements, x, y, z}
$end

$rem
   UNRESTRICTED      false
   METHOD            PBE0
   BASIS             cc-pVTZ
   SYMMETRY false
   SYM_IGNORE true
   PURECART 11111
   MAX_SCF_CYCLES   500
   SCF_ALGORITHM    DIIS_GDM
   MEM_TOTAL        1000
   MEM_STATIC       100
$end

@@@

$molecule
   read
$end

$rem
   UNRESTRICTED      true
   METHOD            pbe0
   BASIS             cc-pVTZ
   SCF_GUESS         read
   MOM_START         1
   SYMMETRY false
   SYM_IGNORE true
$end
```

```
$occupied
   1:6 8 9
   1:8
$end

$alist
   1
$end

@@@

$molecule
   read
$end

$rem
   UNRESTRICTED      true
   METHOD            pbe0
   BASIS             cc-pVTZ
   SCF_GUESS         read
   MAX_SCF_CYCLES    0
   CIS_N_ROOTS       10
   CIS_TRIPLETS      false
   TRNSS             true
   TRTYPE            3
   N_SOL             1
   SYMMETRY false
   SYM_IGNORE true
$end

$alist
   1
$end
```
